# Rapid Reactivation of Extralymphoid CD4 T Cells during Secondary Infection

**DOI:** 10.1371/journal.pone.0020493

**Published:** 2011-05-27

**Authors:** Timothy J. Chapman, Kris Lambert, David J. Topham

**Affiliations:** Department of Microbiology and Immunology, David H. Smith Center for Vaccine Biology and Immunology, Aab Institute of Biomedical Sciences, University of Rochester, Rochester, New York, United States of America; University of California Los Angeles, United States of America

## Abstract

After infection, extralymphoid tissues are enriched with effector and memory T cells of a highly activated phenotype. The capacity for rapid effector cytokine response from extralymphoid tissue-memory T cells suggests these cells may perform a ‘sentinel’ function in the tissue. While it has been demonstrated that extralymphoid CD4+ T cells can directly respond to secondary infection, little is known about how rapidly this response is initiated, and how early activation of T cells in the tissue may affect the innate response to infection. Here we use a mouse model of secondary heterosubtypic influenza infection to show that CD4^+^ T cells in the lung airways are reactivated within 24 hours of secondary challenge. Airway CD4^+^ T cells initiate an inflammatory cytokine and chemokine program that both alters the composition of the early innate response and contributes to the reduction of viral titers in the lung. These results show that, unlike a primary infection, extralymphoid tissue-memory CD4^+^ T cells respond alongside the innate response during secondary infection, thereby shaping the overall immune profile in the airways. These data provide new insights into the role of extralymphoid CD4^+^ T cells during secondary immune responses.

## Introduction

In recent years it has become clear that extralymphoid tissues play an important role in the adaptive immune response by harboring primed T cells following infection or immunization, many of which have an effector phenotype [1], [2], [3], [4]. In animal models of viral infection, effector-memory cells can be recovered from non-lymphoid tissues for several months. As a result, it has been proposed that these cells accumulate in extralymphoid tissues to act as a first line of defense in case of secondary challenge with the same or antigenically related virus [5], [6]. Indeed, it has been shown that primed T cells can be very cross-reactive between infections, suggesting an even more plausible role for extralymphoid cells in site-specific immune protection [7], [8]. In support of this model, several recent reports have demonstrated that extralymphoid memory T cells can be reactivated by secondary infection, and contribute to immune protection [9], [10], [11], [12].

In particular, we recently described a unique population of tissue-memory CD4^+^ T cells recovered from the lungs of influenza immune mice [4]. These local memory cells could be distinguished from their effector memory counterparts in both function and cell surface phenotype, including the expression of the integrin VLA-1. In earlier work, we demonstrated that inhibition or deletion of the alpha chain of VLA-1 leads to a substantial loss of immune protection from secondary challenge [13]. While we attributed most of this effect to a decrease in virus-specific CD8^+^ T cells in the airways, we did not address the contribution of airway memory CD4^+^ T cells to the protective response. Given the recent identification of a highly functional VLA-1^+^ tissue-memory CD4^+^ T cell subset, and the importance of having memory T cells in the airways in models of secondary influenza challenge, we sought to better understand the *in vivo* contributions and behavior of these local memory T cells.

Extralymphoid CD4^+^ T cells may play several roles in their contribution to secondary immunity, ranging from acting as helper cells in the tissue for induction of a protective extralymphoid CD8 response [12] to a direct role as effector cells in elimination of virus [14]. While these studies confirmed the ability of extralymphoid CD4^+^ T cells to respond during secondary infection, they were focused on time points several days after the start of the infection, making it unclear whether the observations reflect the contributions of recently recruited cells from either the circulation or lymphoid sites, or instead reflect the action of those memory T cells in the tissue at the start of infection.

Woodland and colleagues proposed a phased model of the secondary adaptive immune response, consisting of a rapid and early response from tissue-resident cells, followed by accumulation of antigen non-specific cells drawn into the tissue via inflammation, and then accumulation of expanded secondary effector cells prior to pathogen clearance [15]. If this model were correct, the initial phase would be occurring simultaneous to the early innate immune response, leaving open the possibility that there is interaction between the two. A recent report by the Swain lab has furthered our understanding of the early tissue response by demonstrating CD4-dependent alterations in the lung innate inflammatory cytokine milieu in the first two days of secondary influenza infection [16]. While the effect of memory cells on lung innate cytokines was antigen-dependent, the resulting ‘protective state’ induced in the tissue was independent of infection or PAMP recognition, suggesting memory CD4^+^ T cells in the lung were able to orchestrate the early innate response.

Here, we used a model of heterosubtypic influenza infection to study the reactivation of CD4^+^ T cells present in the lung airways at the time of secondary challenge. Our results show that it takes at least three days for newly expanded secondary T cells to arrive in the airway. In contrast, airway CD4^+^ T cells are reactivated within 24 hours of secondary challenge. Infection induces transcriptional changes in airway CD4^+^ T cells, including the up-regulation of a panel of inflammatory chemokines and cytokines, as well as the anti-inflammatory cytokine IL-10. Early innate responses are consequently altered compared to primary infection, likely due to the local early adaptive response. These results demonstrate the sentinel function of airway memory CD4^+^ T cells during secondary influenza infection, and suggest that the combined effects of early innate and adaptive tissue responses contribute to secondary immune protection.

## Results

### Airway memory CD4^+^ T cells decline in numbers but express CD69 during early secondary infection

**Figure 1:**
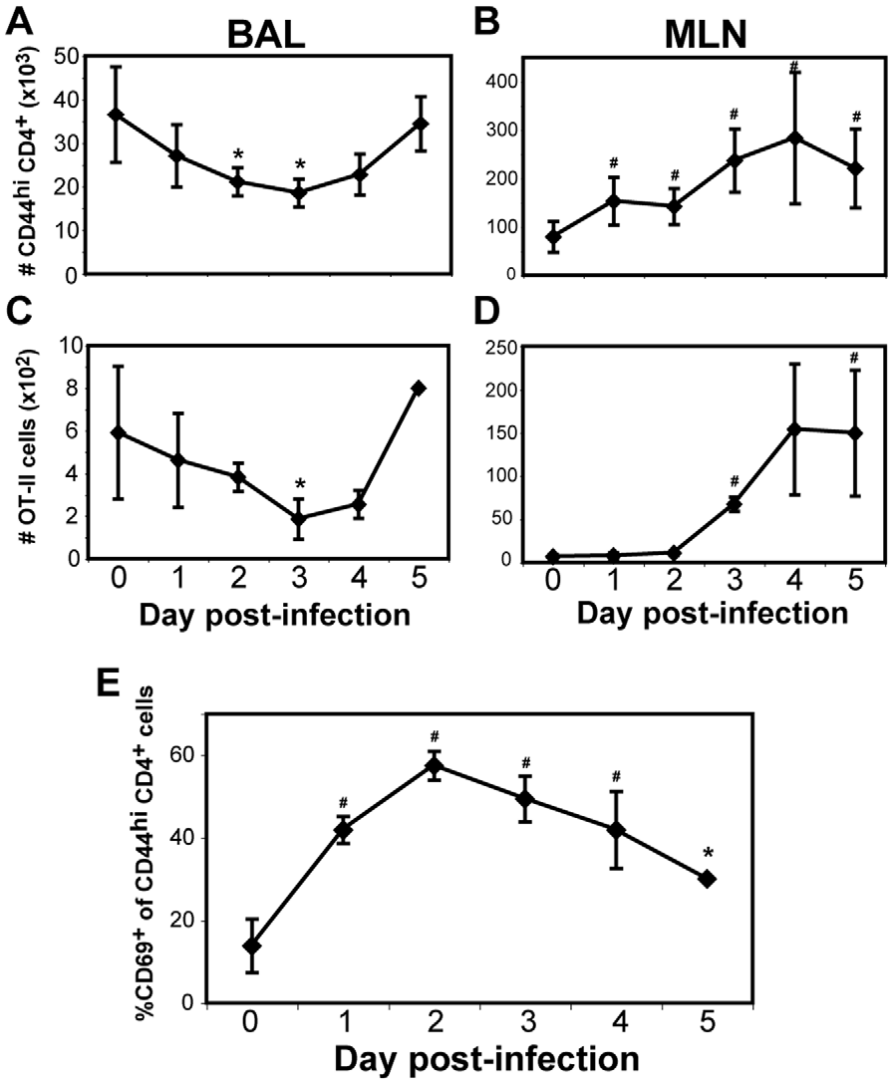
Kinetics of CD4^+^ cell recovery and CD69 expression during secondary influenza infection. OT-II cells were intravenously transferred to mice that were subsequently infected with H1N1 WSN-OVA_II_. One month after infection, a secondary infection with H3N2 X-31/OVA_II_ was given to a cohort of recovered mice. At several time points post-infection, BAL and MLN samples were collected, and CD4^+^ T cells identified as CD44^hi^ CD4^+^ T cells within the lymphocyte gate. OT-II cells identified as CD4^+^ Thy1.1^+^. A–D) Recovery of CD4^+^ T cells after secondary infection. Graphs show CD44^hi^ CD4^+^ (A–B) or CD4^+^ Thy1.1^+^ (C–D) cells from BAL (A, C) or MLN (B, D). E) Percentage of BAL CD44^hi^ CD4^+^ T cells expressing CD69 during secondary infection. Data are representative of three experiments, +/−SEM of n = 3–12 per point. * = p<0.05; # = p<0.01 compared to day 0 data within same group.

In order to study the kinetics of early secondary infection, we utilized a mouse model of heterosubtypic immunity with recombinant influenza viruses that contain the OVA_323–339_ epitope recognized by TCR transgenic OT-II cells [17], [18]. This allowed the parallel monitoring of primed endogenous CD4^+^ and OT-II cells during secondary infection. Our initial prediction was that CD4^+^ cell recovery by BAL would rapidly increase as infection-induced inflammation caused the recruitment of secondary effector cells. Surprisingly, we measured a decline in the recovery of CD44^hi^ CD4^+^ T cells by BAL in the first three days of secondary infection that did not rebound until day 5 (Fig. 1A). The recovery of OT-II cells in the first five days of secondary infection also followed similar kinetics (Fig. 1C). Interestingly, we observed an increase in the proportion of CD69^+^ cells in the BAL as early as 24 hours post-infection, and nearly 60% of BAL CD4^+^ T cells were CD69^+^ at 48 hours (Fig. 1E). This finding raised the question whether the CD69^+^ cells were newly recruited from circulation, or airway tissue-memory cells responding to infection. To address this, we compared the phenotype, function and proliferation of CD4^+^ T cells in various organs during secondary infection.

### CD69^+^ CD4^+^ T cells are present in the airways before the accumulation of proliferating cells

**Figure 2:**
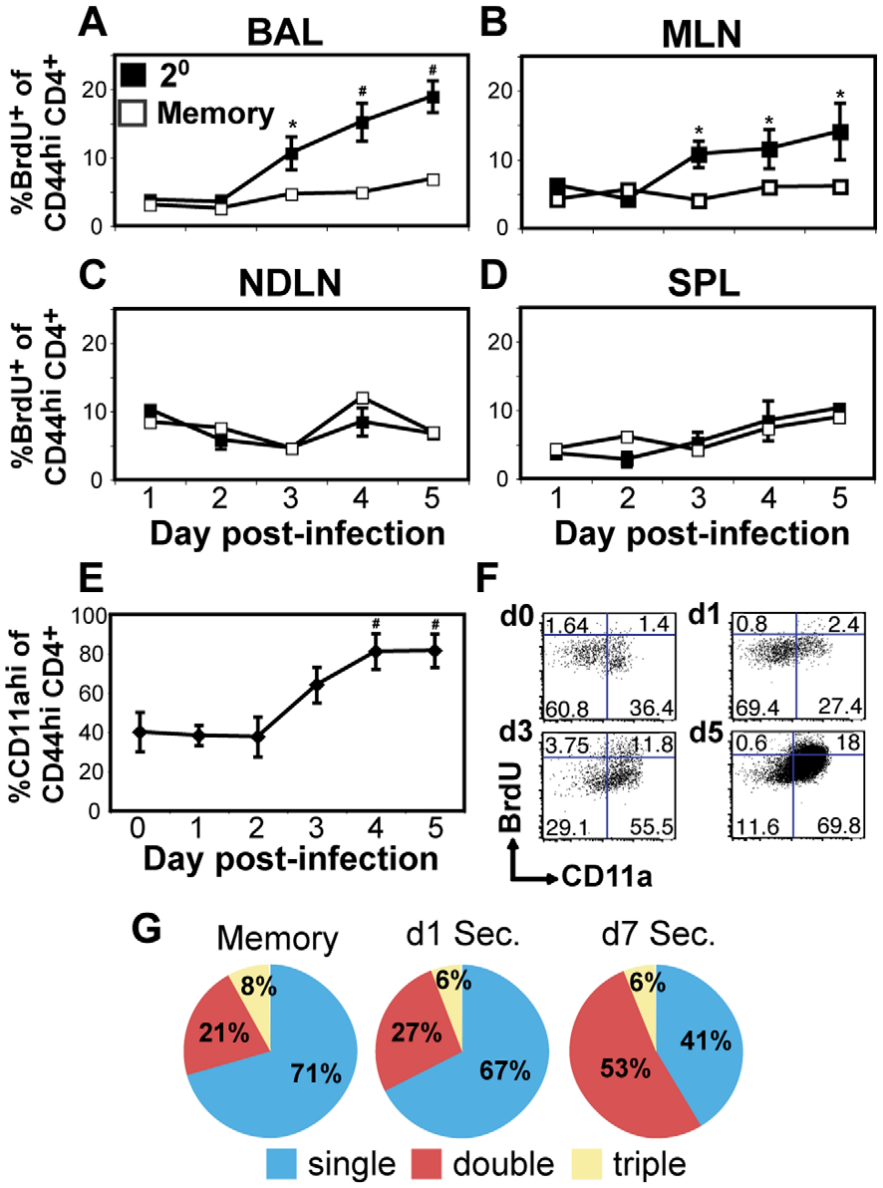
Proliferation and phenotype of memory CD4^+^ T cells during secondary infection. A–D) Mice were infected with WSN-OVA_II_. At one month post-infection, half of the mice were given heterosubtypic secondary infection, and the other half left uninfected. On the day of secondary infection, both memory and secondary mice were administered BrdU i.p., and supplemented with BrdU water thereafter. Mice were sacrificed at several time points, and cells were recovered from BAL, MLN, non-draining lymph node (NDLN) and spleen (SPL). Data shown are the proportion of BrdU^+^ cells amongst the CD44^hi^ CD4^+^ cell subset in either secondary (black) or memory (white) mice. * = p<0.05; # = p<0.01 compared to day 1 values within same group. E) Percentage of BAL CD44^hi^ CD4^+^ T cells expressing CD11a during secondary infection. # = p<0.01 compared to day 0. F) Representative two-dimensional plots of CD11a and BrdU staining of BAL CD44^hi^ CD4^+^ T cells on day 0 (d0), 1, 3, and 5 post-secondary infection. G) Pie charts depicting multiple cytokine producing response of BAL CD44^hi^ CD4^+^ T cells from memory or secondary mice producing IL-2, IFN-γ and/or TNF-α following *ex vivo* re-stimulation. Data are representative of two experiments, +/−SEM of n = 4–5 individual mice per experiment (n = 8–10 total).

Throughout the first five days of secondary infection, there was a modest three-fold increase in CD4^+^ cell recovery from the draining mediastinal lymph node (MLN) (Fig. 1B). In contrast, OT-II cell recovery from MLN increased by ∼20-fold (Fig. 1D), which was consistent with published results of the secondary expansion potential of memory CD4+ T cells [19]. To measure proliferation and airway accumulation of CD4^+^ T cells in response to secondary infection, we used two approaches. The first was to pulse recovered animals with 5′-bromodeoxyuridine (BrdU) and monitor the appearance of divided CD4^+^ T cells in various organs during secondary infection. Figure 2 shows that the proportion of BrdU^+^ CD4^+^ T cells in all organs studied did not increase above that seen in unchallenged memory animals until three or more days after secondary challenge. On day 3 forward, the proportion of BrdU^+^ CD4^+^ T cells in the MLN increased, coinciding with the recovery of BrdU^+^ cells by BAL (Fig. 2A, B). We interpret these observations to indicate that there is limited if any secondary expansion of reactivated CD4^+^ memory T cells in the airway, and that CD4^+^ memory cells from the draining lymph node begin expanding from day 2 forward before they reach the lung.

### Airway memory cells present at the time of secondary infection are phenotypically distinct from the subsequent influx of circulating cells

The second approach to studying cellular dynamics, particularly in BAL, took advantage of some of the unique properties of airway T cells. First, T cells in the airways are continually replaced by circulating memory cells at a steady rate [20]. Second, activated LFA-1^hi^ T cells down-regulate both chains of the integrin LFA-1 (α_L_/CD11a and β2/CD18) in a time-dependent manner after migration to the airways [20]. One observation that is a consequence of this phenomenon is that primed CD4^+^ T cells recovered by BAL one month post-infection have a uniquely broad distribution of CD11a expression (Fig. S1), presumably reflecting a mixture of recently recruited CD11a^hi^ cells as well as memory T cells that have been there longer and have progressively lost integrin expression as a function of time in the tissue. During a rapid influx of T cells from the circulation, we would expect to recover a greater proportion of CD11a^hi^ cells by BAL compared to unchallenged memory animals. Interestingly, the proportion of CD11a^hi^ cells in the BAL remained unchanged for the first 48 hours of secondary infection (Fig. 2E), even though CD69 increased (Fig. 1E) among these cells. It was not until day 3 on that CD4^+^ T cells with a CD11a^hi^ phenotype began to predominate, a time consistent with the appearance of BrdU^+^ cells in both BAL and MLN. Indeed, two-dimensional plots of BrdU and CD11a showed that almost all BrdU^+^ CD4^+^ T cells recovered by BAL were CD11a^hi^ (Fig. 2F). Throughout secondary infection, all CD4^+^ T cells in the spleen were CD11a^hi^ (Fig. S1). These observations reinforce the idea that CD4^+^ tissue-memory T cells are reactivated in the airways, but newly recruited cells from outside the airways take three or more days to appear.

We also compared the effector cytokine profile of memory and secondary CD4^+^ T cells by detection of IFN-γ, IL-2 and TNF-α following *ex vivo* restimulation. BAL samples from resting memory and re-challenged mice were co-cultured with peptide-loaded splenic APCs from naïve mice in the presence of Brefeldin A. Intracellular accumulation of IFN-γ, IL-2 and TNF-α was detected simultaneously in responder cells via multicolor flow cytometry. Single cytokine producers were identified as making only one of the three cytokines, while double producers made any combination of two out of three. The frequency of single, double, and triple cytokine-producing CD4^+^ T cells from the airways was similar in cells recovered from unchallenged memory animals and one day after secondary challenge (Fig. 2G). However, a greater proportion of double cytokine-producing cells were recovered by BAL seven days post-infection (Fig. 2G), suggesting that resident memory cells and those from the influx of secondary effector cells in the circulation differ in their composition of effector functions. Taken together, these data demonstrate that at least two distinct phases of the secondary response in the lung airways are separable based on kinetics, cell phenotype and turnover. The first phase largely consists of CD4^+^ T cells present in the airways at the time of infection. Significant increases in CD69 expression occur in this population within 24 hours of secondary infection, and limited proliferation is observed. In contrast, the second phase is dominated by CD11a^hi^ cells, many of which have divided. Our results do not necessarily distinguish whether these cells begin dividing locally or in the lymphoid tissues, and recent reports suggest that some local proliferation may be possible [11], [21]. In either event, we see no evidence of expansion of CD4^+^ T cells in the airways until day 3 forward.

### Airway tissue-memory CD4^+^ T cells respond to secondary infection within 24 hours

**Figure 3:**
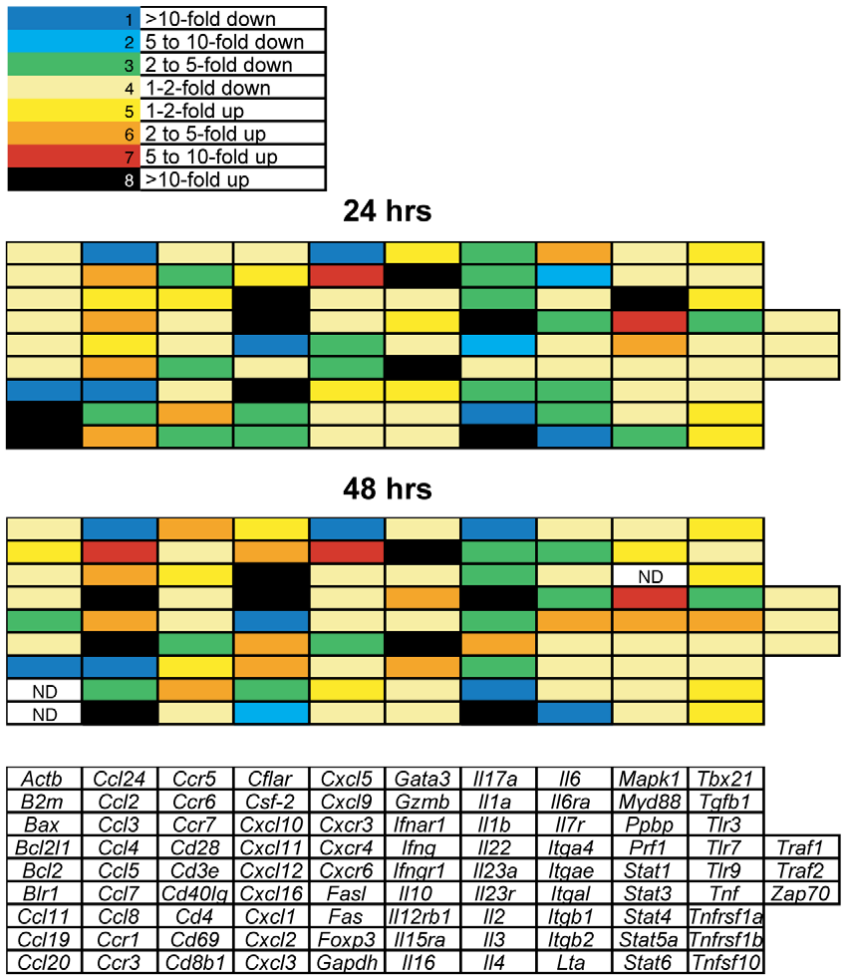
Transcriptional changes in BAL CD4^+^ T cells during early secondary infection. Mice were given heterosubtypic infection as in Figure 1. Another group of mice received primary infection but no secondary. At one or two days post-infection, BAL samples were pooled, and CD3^+^ CD44^hi^ CD4^+^ T cells were FACS sorted to >99% purity. RNA was isolated from sorted samples, and RT-PCR was used in a custom array format to measure gene transcription. The data shown are heat map results comparing 24 hr and 48 hr time points with memory BAL. Fold change in relation to memory BAL is segmented into colors, and the color legend is described at the top of the figure. Each rectangle represents an individual gene, and the heat map legend is at the bottom of the figure. Results are an average of n = 4–5 per group per time point; each n is a pool of BAL from 25–30 mice.

The identification of a distinct population of airway memory CD4^+^ T cells present during the first two days of secondary infection allowed us to determine whether this population was reactivated by infection. To test this, we highly purified endogenous airway CD4^+^ T cells from a cohort of mice that were either challenged with a secondary influenza infection or left unchallenged. After flow cytometric sorting, we analyzed RNA expression of multiple genes by RT-PCR. The total dataset is in Figure 3, and the subset of genes that were significantly different between memory and secondary airway CD4^+^ T cells are in Table 1. Compared to cells from the unchallenged mice, airway CD4^+^ T cells recovered within 24 hours of secondary infection had induced a variety of inflammatory genes consistent with their reactivation to infection (Fig. 3 and Table 1). The profile of induced genes was roughly what would be expected for a T_H_1 cell response in the airway, including genes for IFN-γ inducible chemokines and cytolytic granule proteins. However, we also found a concomitant increase in the anti-inflammatory cytokine IL-10 (Table 1). These data suggest reactivated T_H_1 cells may self-regulate inflammation via IL-10 secretion in a similar fashion as airway CD4^+^ T cells that are present during the resolution of primary influenza infection [22]. Alternatively, distinct populations of CD4^+^ T cells may be responding during the early airway response, focused both on directing antiviral inflammation and reducing pathology.

**Table 1:** BAL CD4+ cell transcriptional changes after secondary infection.*

24 hours			48 hours		
Gene	Product	Fold change#	Gene	Product	Fold change#
Cxcl10	CXCL10	117.4	Gzmb	Granzyme B	75.1
Gzmb	Granzyme B	32.2	Cxcl10	CXCL10	52.5
Cxcl11	CXCL11	31.7	Il10	IL-10	38.1
Cxcl9	CXCL9	15.3	Cxcl11	CXCL11	29.1
Il10	IL-10	11.7	Ccl4	CCL4	21.1
Ccl4	CCL4	5.9	Ccl7	CCL7	20.7
Prf1	Perforin	3.3	Cxcl9	CXCL9	16.1
Il12rb1	IL-12Rβ1	3.3	Ccl2	CCL2	11.8
Stat1	STAT1	2.9	Ccl3	CCL3	6.5
Tnfsf10	TRAIL	1.6	Prf1	Perforin	4.6
Ifngr1	IFN-γRI	−1.5	Il12rb1	IL-12Rβ1	3.7
Cxcr3	CXCR3	−1.6	Bcl2	BCL-2	−2.5
Bax	BAX	−1.8	Cxcl12	CXCL12	−123.0
Itgb1	Integrin β1	−1.9			
Bcl2	BCL-2	−2.0			
Tnfrsf1a	TNFRI	−2.0			
Itgb2	Integrin β2	−2.0			
Il23	IL-23	−2.2			
Itga4	Integrin α4	−2.3			
Cxcr6	CXCR6	−2.7			
Cd40Ig	CD40L	−3.0			
Cxcl12	CXCL12	−121.2			
Ccl24	CCL24	−756.1			
Il3	IL-3	−3952.7			

*Identified as p<0.01 compared to memory BAL values (see Materials and Methods).

# Fold change in secondary BAL CD4+ gene expression compared to memory.

**Figure 4:**
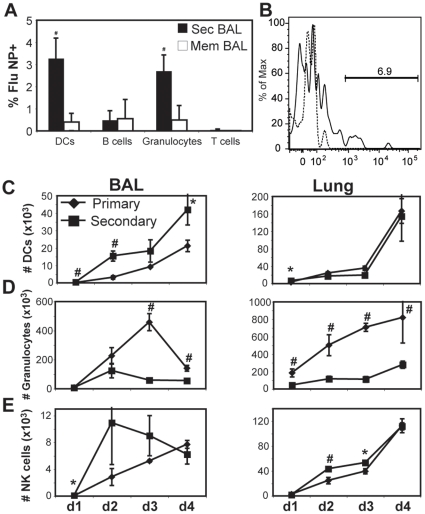
Comparison of primary and secondary innate responses in the lung. A) BAL cells from memory (white bars) or 18 hr post-secondary (black bars) mice were stained intracellularly with antibody to influenza nucleoprotein (NP) on dendritic cells (MHC class II^+^ CD11c^+^), B cells (CD11c^−^ CD11b^−^ Class II^+^ B220^+^), Granulocytes (Gr-1^+^ CD11b^+^) and lymphocytes (CD11c^−^ Gr-1^−^ CD3ε^+^). Percentages indicate the proportion of each population that was NP-positive. # = p<0.01 compared to memory data within same group. B) Staining for NP in the CD11c^+^ MHC Class II^+^ cells collected from memory (dashed line) and secondarily infected (solid line) BAL. C–E) Cell recovery of MHC Class II^+^ CD11c^+^ cells (C), Gr-1^+^ CD11b^+^ cells (D), and NK1.1^+^ DX5^+^ CD11c^+^ CD3ε^−^ MHC Class II^−^ cells (E) from BAL and lung of mice after primary (diamonds) or secondary (squares) infection. The average NK cell numbers for day 1 are 11 and 68 for primary and secondary, respectively, with p = 0.02. The average BAL DCs on day 1 are 55 vs. 348, p = 0.006; and lung is 3200 vs. 6400, p = 0.03. Data are representative of 3 experiments, +/−SEM of n = 3–5 per data point. * = p<0.05; # = p<0.01 comparing primary and secondary data on same day.

The rapid reactivation of CD4^+^ T cells in the airways raised the question as to the nature of the re-activating antigen-presenting cell. Recent published data has shown that antigen presentation in the lung provides an important activating signal for effector T cells during primary infection [23], [24]. In addition, data from the Heath lab suggests a CD4-CD8-dendritic cell interaction can occur in the infected tissue for re-activation of local T cells [12]. We therefore studied the innate cells recovered by BAL 18 hours post-secondary infection, and used an antibody to influenza nucleoprotein as a means of detecting cells carrying intracellular viral antigen. We found Gr-1^+^ and CD11c^+^ cells were the two major cell types from BAL that stained positive for viral nucleoprotein (Fig. 4A–B). Since about two-thirds of the recovered CD11c^+^ cells were MHC class II^+^ and <1% of Gr-1^+^ cells were class II^+^ (not shown), these data suggest CD11c^+^ lung dendritic cells are likely capable of presenting viral antigens to local CD4^+^ T cells during the early stage of secondary infection.

### The composition of the lung innate cell response is altered during secondary infection

One contribution of virus-specific memory T cells in the airways is a modification of the local inflammatory response [16]. In further analysis, we discovered profound differences in the recovery of several lymphoid and myeloid cell populations from the airways comparing primary and secondary influenza infection (Fig. 4C–E). In the early stages of infection, there was increased recovery of CD11c^+^ Class II^hi^ cells and CD3^−^ NK1.1^+^ cells compared to primary infection, while total Gr-1^+^ cells were reduced, and CD11b^+^ CD11c^−^ cells were unchanged (Fig. 4 and data not shown). In further analysis of airway CD11c^+^ Class II^hi^ cells, there was an increased proportion of cells expressing CD80/86, as well as increased recovery of both CD11b^+^ CD103^−^ and CD11b^−^ CD103^+^ dendritic cell populations 24 hr after secondary infection compared to primary (Fig. S2) [25]. These differences in innate cell recovery are likely independent of lung conditioning by the primary infection, since mice given intranasal LPS treatment (as a means of conditioning the lung) prior to influenza had similar innate cell recoveries as mice given primary infection but not re-challenged (not shown).

### Role of CD4^+^ T cells on the composition of the innate response and lung viral titer during secondary infection

To assess whether CD4^+^ T cells were important for the observed changes in innate cell recovery, we treated immune memory animals with GK1.5 antibody to CD4 prior to secondary challenge. This resulted in a complete depletion of CD4^+^ T cells from lymphoid organs and substantial, but not complete depletion from the lung (data not shown). During a 4-day period after infection, we observed modest changes in the recovery of innate cells by BAL in GK1.5-treated mice compared to controls that were intermediate in value between mice with primary infection or non-depleted controls (Fig. S3), suggesting local tissue-memory CD4^+^ T cells may alter the inflammatory cell milieu and play a role in shaping the early innate response. However, since depletion of CD4^+^ T cells from the lung was incomplete, we cannot rule out the contribution of residual CD4^+^ memory T cells and/or a level of redundancy between the airway CD4^+^ and CD8^+^ tissue memory cell responses.

**Figure 5:**
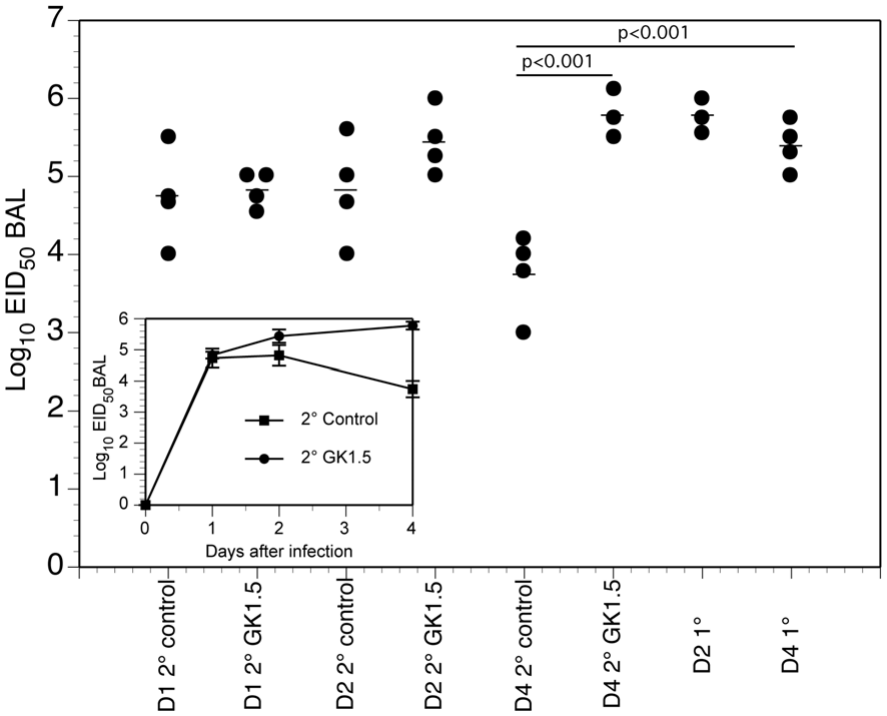
Impact of CD4^+^ T cells on viral titers during secondary infection. BAL supernatants from memory, secondary infected, and GK1.5-treated secondary infected mice were injected into embryonated chicken eggs for determination of viral titers (see Materials and Methods). Samples from day 1, 2, and 4 post-secondary infection (with X-31 virus) were compared +/− GK1.5 treatment, with primary X-31 infection as control. Mean and SEM from the n = 3–4 mice per time points per group are indicated by the horizontal bar symbols and in the inset figure. A Kruskal-Wallis rank test was performed, with significant p values shown.

In order to compare GK1.5 and control-treated animals in their capacity to control infection, viral titers were measured from BAL supernatants of primary and secondary infected lungs. Viral titers in GK1.5 and control-treated BAL were comparable one and two days after secondary infection, with a trend towards an increased titer in GK1.5-treated animals on day two (Fig. 5). On day four, there was a significant decrease in viral titer in secondary infected control mice compared to primary infection, and this decrease in viral titer was lost in GK1.5 treated mice (Fig. 5), suggesting CD4^+^ T cells contribute to the rapid viral clearance observed between primary and secondary infection.

## Discussion

In summary, these studies demonstrate rapid responsiveness of a local population of CD4^+^ T cells in the lung airway at the time of secondary infection. Airway cells respond 24-48 hours prior to the appearance of divided secondary effectors, and turn on an inflammatory response program that promotes reduced viral titers and may help shape the developing innate response. After the first two days of infection, a second phase of divided cells can be detected that differ in both functional and cell surface phenotypes. This report, in conjunction with previous reports demonstrating antigen non-specific accumulation of primed T cells during infection [11], [17], [26], [27], provides further support for the multi-phase model of adaptive immune responsiveness to secondary infection.

In order to understand the overall immune response to secondary contact with a pathogen, it is important to first identify when and where the different populations of immune cells participate in the response. Here we have carefully documented the reactivation, timing, phenotype, and gene expression profiles of the flu-specific memory CD4^+^ T cells in the airways. We have previously shown that the CD4^+^ T cells in the airways of immune mice are functionally distinct from those in the lymphoid organs and are comprised of at least two populations differentiated by alpha-1 integrin expression and reactivation potential [4]. Mice that are deficient in alpha-1 integrin demonstrate increased susceptibility to secondary virus challenge [13] but have no defects in a primary response to non-lethal influenza challenge. The diminished protection has been associated with a reduction in the number of virus-specific memory T cells present in the lung at the time of re-challenge, and is also observed in wild-type mice after longer time intervals [13], [28], [29], [30]. Although much of this loss of protection has been attributed to cytotoxic CD8^+^ T cells [13], [29], [30], local CD4^+^ memory T cells are also present. The data presented herein shows that these cells are activated and participate in the response, potentially by modifying the inflammatory response and/or directly contributing to reduction in lung viral titer.

One outstanding question not addressed in this report is the proportion of airway memory T cells that respond to secondary infection *in vivo*. While a high proportion of virus-specific T cells respond in *ex vivo* restimulation, whether reactivation is uniform *in vivo* remains to be determined. The specific localization of memory cells within the lung and airway environment may be important. For example, data from our laboratory suggest airway CD4^+^ and CD8^+^ cells expressing the α1β1 integrin VLA-1 may be uniquely positioned for secondary responsiveness due to the capacity of VLA-1^+^ cells to localize near large airways [4], [31]. Presumably, localization within the airway epithelium is a critical feature of rapid and effective immune function. While it is easy to appreciate the potential for cytotoxic CD8^+^ T cells to engage class I MHC on infected epithelial cells, the mechanisms by which CD4^+^ T cells are reactivated remain less clear. While our data suggests that professional antigen-presenting cells bearing influenza antigen are more numerous in immune mice, it is possible that both antigen-specific responses driven via class II MHC interactions, as well as antigen non-specific responses via inflammatory cytokines [11], [32] could result in multiple antigen specific and non-specific T cell populations responding in concert during early infection.

We discovered profound differences in the cellular composition of the lung innate response to secondary infection compared to primary infection. While it seems clear from our data and others [16] that local adaptive memory can shape the early tissue response, it is unclear how these changes in the lung affect the outcome of infection. For instance, in analysis of cytokine secretion from endogenous airway memory CD4^+^ T cells, we found a significant increase in IL-10 expression during secondary infection. This was in association with a decreased recovery of granulocytes compared to primary infection, consistent with published data on Gr-1^+^ cells in IL-10^−/−^ mice after bleomycin-induced lung injury or fungal infection [33], [34]. In addition, aged mice have been shown to have increased levels of IL-10, which appears to reduce innate responses in the lung [35]. These data suggest that increased IL-10 production from CD4^+^ T cells may limit the granulocyte response during secondary infection. However, depletion of memory CD4^+^ T cells in our studies not only alters the contribution of memory CD4^+^ T cells themselves to secondary immune protection, but also the CD4-induced changes in the local innate inflammatory and cellular environment. It would be useful to have a system in which memory CD4^+^ T cells established by respiratory infection are the only cross-reactive T cell subset, though such a system does not currently exist. With such a system, it may be possible to further segregate the relative contributions of adaptive memory and the altered innate response in protection.

Our results complement a recent report showing increased innate inflammatory cytokine secretion in the lung during secondary infection [16] by profiling the contribution of the memory CD4^+^ T cells themselves to the early inflammatory environment, and suggest that airway memory cells present at the time of infection, rather than circulating memory cells recruited to the lung, are responsible for the early changes observed. We also show that, coupled with changes in innate inflammatory cytokines, innate cell recovery is significantly altered by the presence of an adaptive tissue response, and may be the result of CD4^+^ T cell reactivation. We propose that rapidly responding extralymphoid tissue memory cells have the capacity to shape changes in the composition of the local innate response that result in a greater capacity for viral clearance and regulation of immune pathology. Further investigation into the protective and pathogenic roles of extralymphoid memory cells will give greater understanding of their importance in immune protection.

## Materials and Methods

### Animals

C57Bl/6 and B6.SJL mice were purchased from the National Cancer Institute. T cell receptor transgenic OT-II mice [36] on the B6.PL background (gift from Dr. Linda Bradley) were bred and maintained at the University of Rochester Medical Center in specific pathogen-free conditions. All animals were ethically handled in compliance with the University of Rochester Institutional Biosafety Committee guidelines.

### Viral infection

Influenza virus stocks of A/HKx31, A/HKx31/OVA_II_ (gift from Dr. Richard Webby [18]) and A/WSN-OVA_II_ [17] were thawed from −80°C and diluted in sterile PBS prior to use. Recipient mice were first sedated with avertin (2,2,2-tribromoethanol) i.p., then given a 30 µl intranasal inoculation of virus.

### Organ collection

Mice were administered a lethal dose of Avertin by intraperitoneal injection and exsanguinated via the brachial artery. Bronchoalveolar lavage (BAL) samples were collected by three intratracheal lung washes (with C-mem) using a Teflon canula attached to a 1 ml syringe. Lung tissue, MLN and spleen were removed separately. For lymphocyte analysis, BAL samples were subjected to 45 min. plastic adherence prior to use; for innate cell analysis, this step was skipped. Lung tissue was minced with scissors, then ground in a tea strainer. Resulting homogenate was incubated with 0.05% type IV collagenase (Sigma) and 0.003% DNase I (Sigma) for 60 min. at 37°C. Cells were washed, re-suspended in HBSS, and lymphocytes isolated by Histopaque 1083 (Sigma) underlay and centrifugation. MLN and spleen were homogenized and filtered through nylon mesh. All organ cells were maintained in C-mem prior to use.

### Flow cytometry

The following antibodies were used in surface and intracellular staining procedures: IL-2/FITC, NK1.1/FITC, IFN-γ/PE, CD69/PerCP-Cy5.5, CD11a/PE-Cy7, CD11c/PE-Cy7, TNF-α/APC, BrdU/APC, B220/APC, Gr-1/APC-Cy7 and CD11b/biotin purchased from BD; MHC Class II I^A^/I^E^/PE-Cy5, CD62L/APC-Cy7 and CD4/Alexa 700 purchased from eBioscience; DX5/PE and CD44/Pacific Blue purchased from Biolegend; streptavidin-Pacific Orange purchased from Invitrogen. *Surface staining:* 1-2×10^6^ cells from each organ were placed in individual wells of a 96-well round-bottom plate for staining. Fc receptors were blocked with anti-CD16/32 (clone 2.4G2, from BD) for 15 min. Cells were washed and surface stained with various antibodies from the above list, diluted in PBS/BSA and incubated for 30 min. at room temperature. Cells were washed before analysis.

#### Intracellular staining

spleen cells from naïve B6.SJL (CD45.1^+^) mice were used as APCs and infected with influenza (MOI = 1) in 1 ml serum-free media for 60 min. Infected cells were then washed and resuspended in C-mem. 1×10^6^ APCs were added to 1×10^6^ responders (prepared as described above). Golgi Plug (BD) was then diluted 1 µl/ml in C-mem and 100 µl added to each well. Cells were incubated for 5–6 hr at 37°C. Samples were then surface stained as described above. Samples were washed and resuspended in 100 µl/well Cytofix/Cytoperm (BD) for 15 min. After one Perm/Wash (BD), anti-cytokine antibodies were added in Perm/Wash, and cells incubated for 30 min. on ice in the dark. Samples were resuspended in PBS/BSA for FACS.

#### Detection of incorporated Bromodeoxyuridine (BrdU)

Cohorts of mice were administered 1 mg BrdU per mouse by i.p. injection at day 0. For the duration of the experiment, mice were maintained on BrdU-enriched water at a concentration of 0.5 mg/ml. After cell recovery from animals, followed by surface staining and permeabilization, the APC anti-BrdU kit (BD) was used to detect BrdU^+^ DNA in cells. In short, fixed and permeabilized cells were incubated with Cytoperm Plus buffer to permeabilize nuclei, and then treated a second time with Cytofix/Cytoperm for re-fixation of cells. Cells were then treated with DNase to expose BrdU, and subsequently stained with APC anti-BrdU for detection via cytometry. Gating of BrdU^+^ cells was determined by parallel staining of cells that did not receive BrdU in the experiment as a negative staining control. All FACS was run on an LSRII flow cytometer (BD), and analyzed using FlowJo software (Treestar).

### Transcriptional profiling

For detection of gene transcription, custom microfluidics cards were purchased from Applied Biosystems containing 96 unique genes in a 384-well format.

#### Sample collection

One month after A/WSN infection, a cohort of mice were given an X-31 secondary infection. BAL samples were collected from three groups: memory, 24 hr post-secondary, and 48 hr post-secondary. Pools of BAL cells (BAL pooled from 25–30 mice for each data point) were processed and surface stained. Cells were then sorted with a FACSAria cell sorter (BD) by CD3^+^ CD4^+^ CD44^hi^ staining to >99% purity. Sorted cells were washed, lysed using RLT buffer (Qiagen), spun through Qiashredder columns (Qiagen), and stored at −80°C. All steps were done cold to limit RNA degradation. RNA isolation and quality control was performed by the University of Rochester Functional Genomics core (Rochester, NY). Samples were run using a 7900HT Real time PCR system (Applied Biosystems). Housekeeping genes (Gapdh, 18s RNA, β-actin) were compared between memory and secondary samples to ensure comparable expression between groups. Obtained RQ values for each gene were then compared across replicates and groups to determine fold changes in gene expression. No fold cutoff filter was used after statistical analysis. Data was analyzed using SDS software (ABI) and Excel (Microsoft).

### Viral titers

#### Infection of eggs

BAL supernatants were initially diluted 1∶10 in PBS/gentamycin, and a ten-fold dilution series was made from this. 100 µl of each sample dilution was injected into a ten day incubated viable chicken egg beneath the air pocket. Each sample dilution was done in triplicate. The egg injection site was sealed with wax and the eggs placed in a static incubator at 37°C for 48 hours. The eggs were then removed and placed at 4°C for >24 hours.

#### Hemagglutination assay

50 µl of PBS was added to each well of round-bottom 96-well plates, allowing 2 wells per egg, and including positive control wells of virus on each plate. Using a glass pipette, allantoic fluid was withdrawn from each egg and 100 µl was added to the appropriate wells on the 96-well plate. Concurrently, chicken red blood cells were washed in PBS three times and resuspended at 1∶100 in PBS. 50 µl of diluted chicken RBCs was added to each well. The plates were allowed to incubate for 30 min at room temperature. The wells were read as positive or negative depending on lysis of the chicken RBCs. A calculation was performed to determine EID_50_ for each sample.

### Statistics

#### Standard analyses

For comparison of sets of unpaired data, two-tailed T test was used. Resulting p values <0.05 were considered significant.

#### Analysis of RT-PCR data

Permutation test was used to detect differentially expressed genes in BAL cell populations after secondary infection. Permutation test was performed on the log2 scale, (log2(RQ)) comparing memory BAL to secondary BAL. Taking into account both Bonferroni correction and control of per-family error rate [37] to deal with the inflated study-wide type I error due to multiple comparisons, p values <0.01 were considered significant. For comparisons of cell number and virus titers among the groups, unpaired, non-parametric Wilcoxon or Kruskal-Wallis rank tests were performed.

## Supporting Information

Figure S1 **Comparison of CD11a profiles in BAL and spleen.** Representative histograms depicting CD11a profiles of CD44^hi^ CD4^+^ T cells from BAL and spleen during the first five days of secondary infection. Data are representative of 3 experiments, n = 3–5 per time point.
(TIFF)

Figure S2 **Comparison of airway dendritic cell populations between primary and secondary influenza infection.** 24 hr after primary or secondary influenza infection, cells were recovered and stained by flow cytometry. The proportion of lung Class II^+^ CD11c^+^ dendritic cells expressing both CD80 and CD86 (A), as well as the number of BAL CD11b^+^ CD103^−^ and CD11b^−^ CD103^+^ populations among Class II^+^ CD11c^+^ cells (B) was determined. Data are +/− SEM of n = 4 per group. In (A), # = p<0.01. In (B), * = p<0.05 comparing primary and secondary groups.
(TIFF)

Figure S3 **Effect of GK1.5 administration on secondary lung innate cell recoveries.** A–C) Mice were given a primary WSN infection or nothing. One month post-infection, half of the immune mice were administered GK1.5 to deplete CD4^+^ T cells. All mice were infected with X-31 two days after antibody administration. Innate cell recovery of dendritic cells, granulocytes and NK cells by BAL were determined by flow cytometry as in Figure 4. Data are representative of 2 experiments, +/−SEM of n = 3–5 per data point. *p<0.05 comparing primary to secondary and secondary GK1.5 treated groups.
(TIFF)
