# Supplementary figures and images for: Rapid Reactivation of Extralymphoid CD4 T Cells during Secondary Infection
Source: PLoS One. 2011 May 27;6(5):e20493. doi: 10.1371/journal.pone.0020493 (PMC3103554; doi:10.1371/journal.pone.0020493)

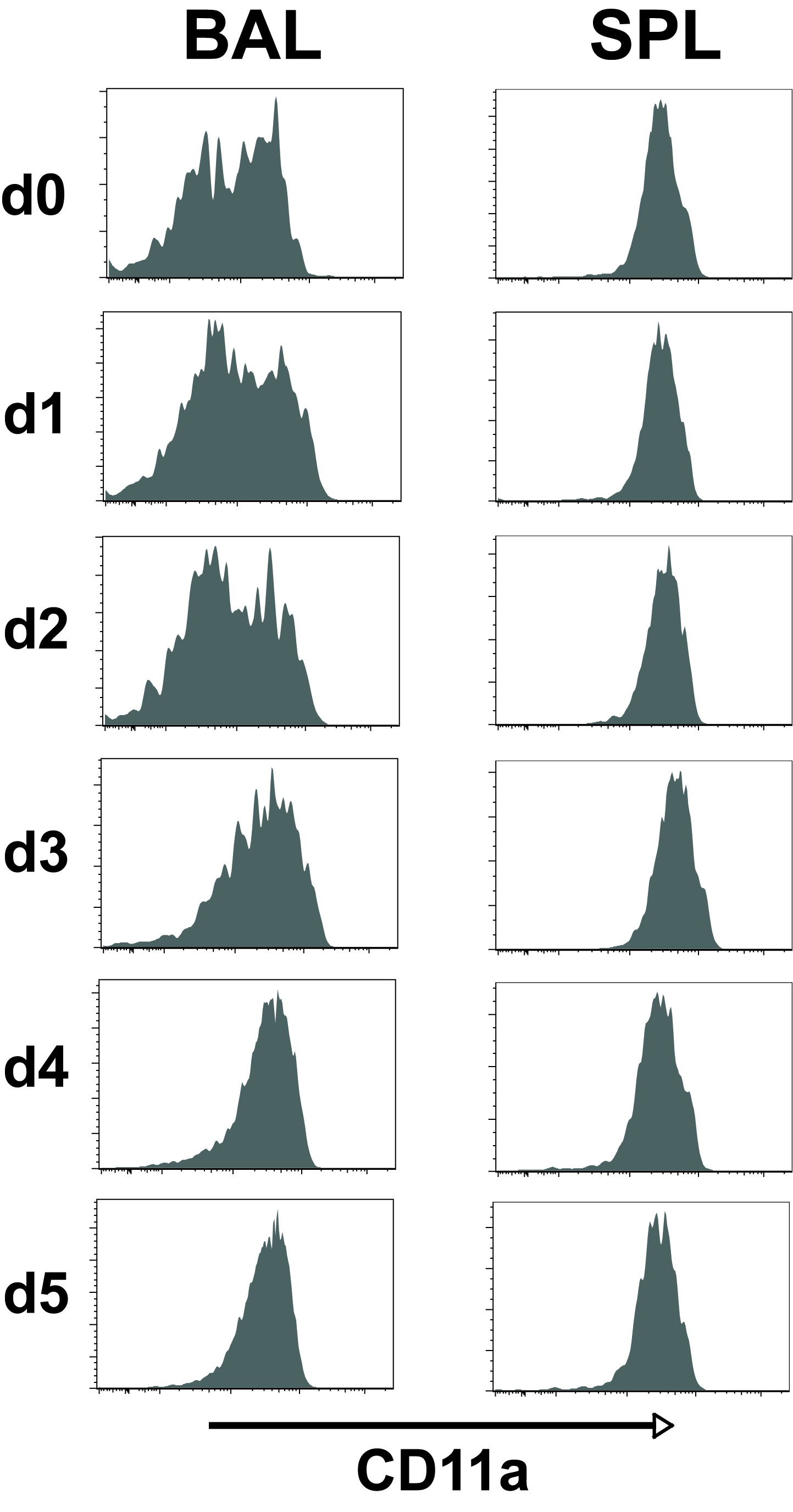

Supplement: Figure S1 — Comparison of CD11a profiles in BAL and spleen. Representative histograms depicting CD11a profiles of CD44hi CD4+ T cells from BAL and spleen during the first five days of secondary infection. Data are representative of 3 experiments, n = 3–5 per time point. (TIFF) [file pone.0020493.s001.tif]

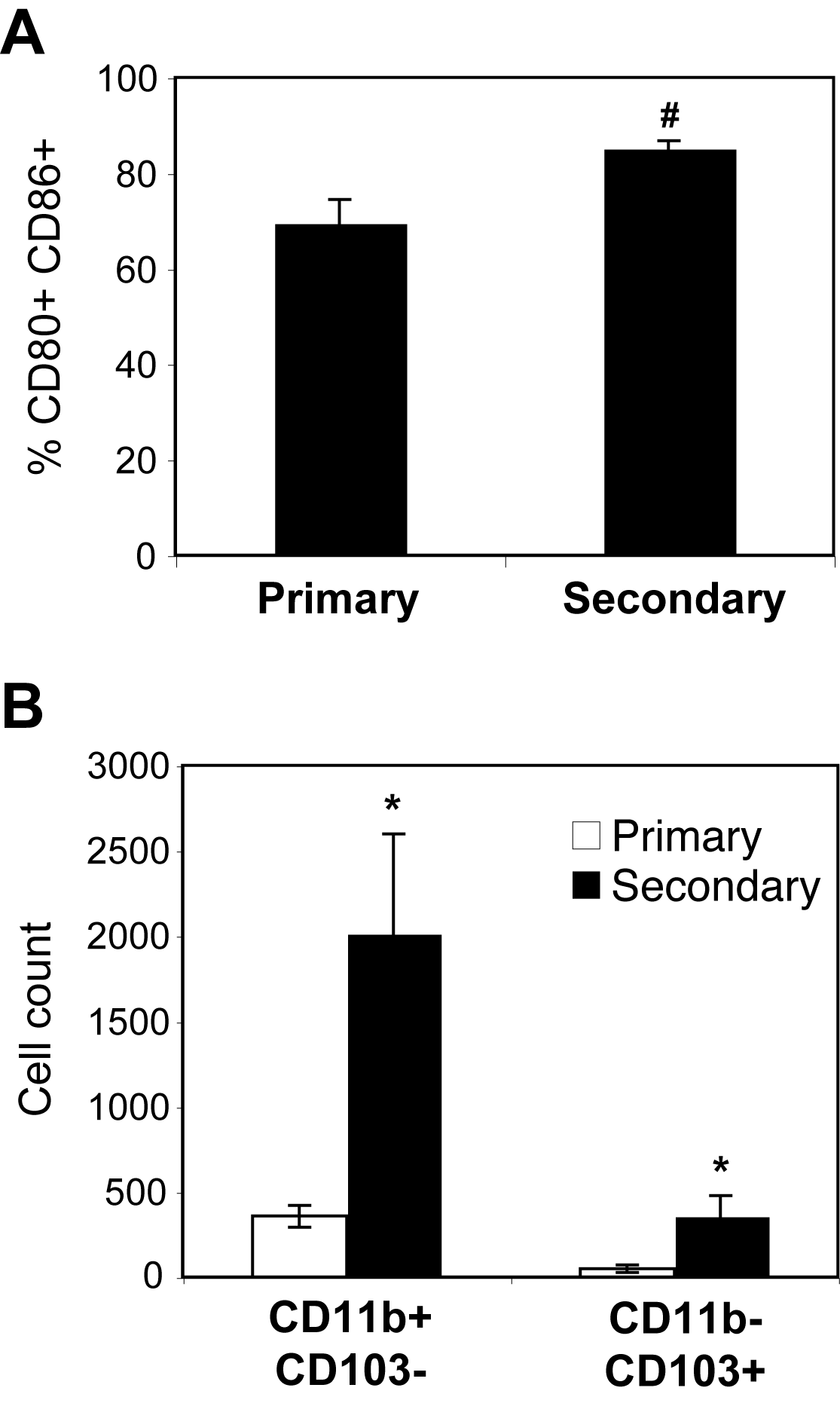

Supplement: Figure S2 — Comparison of airway dendritic cell populations between primary and secondary influenza infection. 24 hr after primary or secondary influenza infection, cells were recovered and stained by flow cytometry. The proportion of lung Class II+ CD11c+ dendritic cells expressing both CD80 and CD86 (A), as well as the number of BAL CD11b+ CD103− and CD11b− CD103+ populations among Class II+ CD11c+ cells (B) was determined. Data are +/− SEM of n = 4 per group. In (A), # = p<0.01. In (B), * = p<0.05 comparing primary and secondary groups. (TIFF) [file pone.0020493.s002.tif]

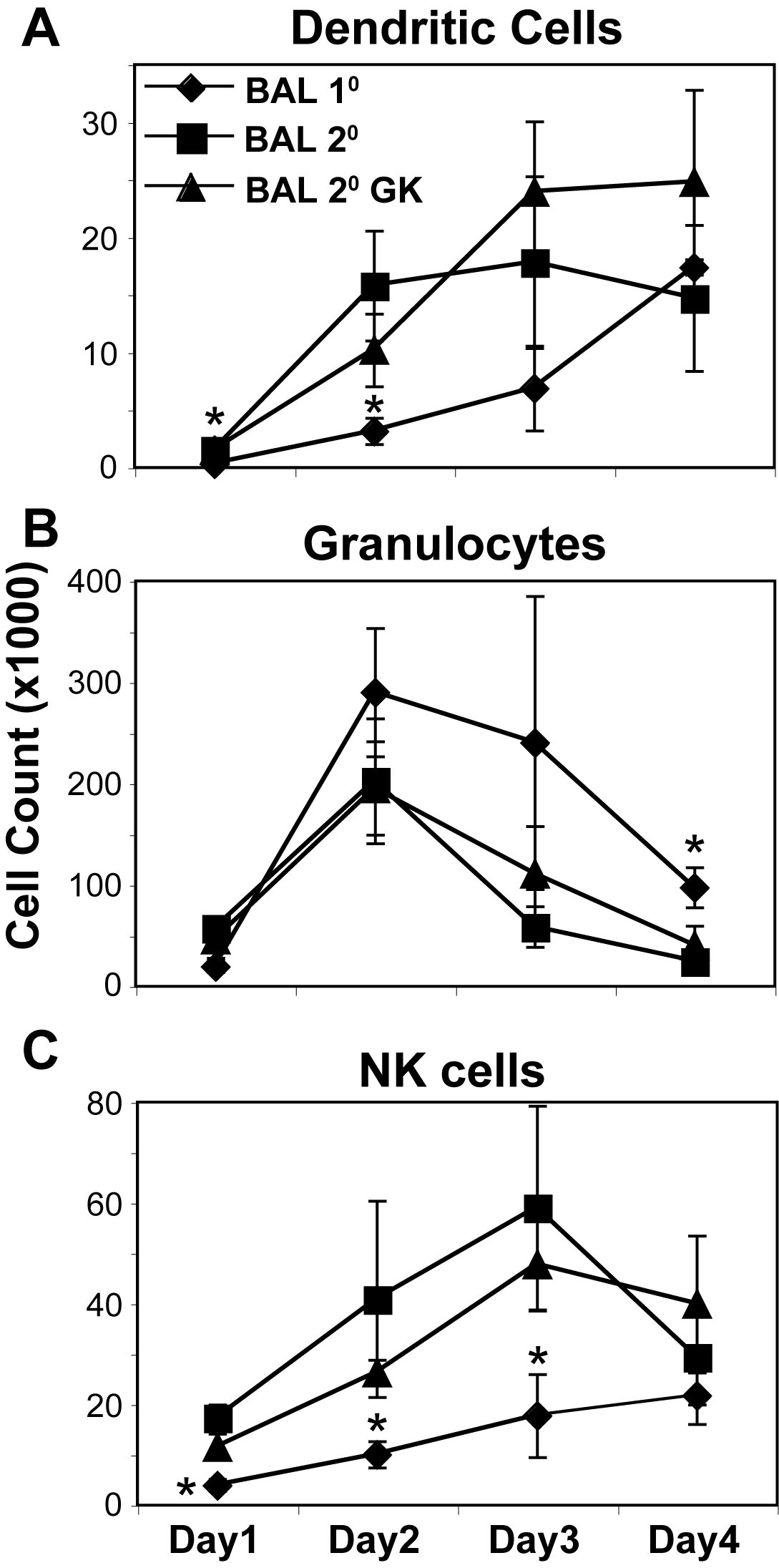

Supplement: Figure S3 — Effect of GK1.5 administration on secondary lung innate cell recoveries. A–C) Mice were given a primary WSN infection or nothing. One month post-infection, half of the immune mice were administered GK1.5 to deplete CD4+ T cells. All mice were infected with X-31 two days after antibody administration. Innate cell recovery of dendritic cells, granulocytes and NK cells by BAL were determined by flow cytometry as in Figure 4. Data are representative of 2 experiments, +/−SEM of n = 3–5 per data point. *p<0.05 comparing primary to secondary and secondary GK1.5 treated groups. (TIFF) [file pone.0020493.s003.tif]
